# Allelopathic Activity of Canadian Goldenrod (*Solidago canadensis* L.) Extracts on Seed Germination and Growth of Lettuce (*Lactuca sativa* L.) and Garden Pepper Cress (*Lepidium sativum* L.)

**DOI:** 10.3390/plants12071421

**Published:** 2023-03-23

**Authors:** Asta Judžentienė, Jurga Būdienė, Linas Labanauskas, Donata Stancelytė, Irena Nedveckytė

**Affiliations:** 1 Center for Physical Sciences and Technology, Department of Organic Chemistry, Sauletekio Avenue 3, LT-10257 Vilnius, Lithuania; 2 Life Sciences Center, Institute of Biosciences, Vilnius University, Sauletekio Avenue 7, LT-10257 Vilnius, Lithuania

**Keywords:** *Solidago* spp., Asteraceae, essential oils, extracts, GC/MS, HPLC/DAD/TOF, allelopathy

## Abstract

Native to N. America, Canadian goldenrod (*Solidago canadensis* L.) was introduced to Europe as an ornamental plant and quickly spread here and in other parts of the world. The rapid spread of the plant is due to several reasons: phenotypic plasticity, broad climatic tolerance, propagation via underground rhizomes and seeds that mature in large numbers, etc. Additionally, the success of Canadian goldenrod’s invasion is determined by its allelochemicals that affect seed germination, root formation and whole growth of nearby plants. Allelopathy of various extracts and essential oils (EOs) of *S. canadensis* on seed germination and growth of lettuce (*Lactuca sativa* L.) and garden pepper cress (*Lepidium sativum* L.) was evaluated and compared with other *Solidago* species (*S. virgaurea, S. × niederederi*) collected from the same growing locality in Lithuania. Soil characteristics (conductivity, pH and major elements) of the collecting site were determined. Aqueous flower extracts of all studied *Solidago* species showed the highest inhibitory effect on model plants. Canadian goldenrod leaf water/diethyl ether extract showed highest inhibitory effect in all relative concentrations (1.0; 0.1; 0.01) suppressing growth of *L. sativa* (from 0 to 2.3 mm compared with 22.7 mm for control samples) and *L. sativum* (from 0.5 to 16.8 mm compared with 35.3 mm in control). It was noticed that garden pepper cress was more susceptible to *Solidago* spp. inhibitory effects than lettuce. *S. canadensis* root EOs comprised mainly of limonene (35.0%) and β-pinene (26.2%) and inflorescence oils containing α-pinene (21.6%), germacrene D (15.1%), limonene (10.2%) and lupenyl acetate (9.8%) exhibited the highest inhibitory effect on lettuce and garden pepper cress growth. Relative germination and vigor index of model plants was conducted. Chemical composition of extracts and EOs was determined by HPLC/DAD/TOF and GC/MS techniques.

## 1. Introduction

There are about 140 species of *Solidago* L. (Asteraceae) in the world, of which 115 are native to North America, around 8 species originate from Mexico, and about 13 species are indigenous to South America, the Azores, Europe and Asia [1]. All *Solidago* species found in local flora or cultivated as ornamentals are herbaceous and rhizomatous perennials (up to 2.5 m in height) with yellow ray florets.

*Solidago canadensis* L., commonly known as Canadian goldenrod, native to N. America is widely spread throughout Europe, Asia (China, Russia, Japan, and Taiwan), Australia and N. Zealand, where it is considered an aggressive invasive weed [2,3,4]. The taxon is highly diverse, prone to hybridization, and therefore characterized by extensive polyploidy [1,2,5,6,7,8]. It is known that two N. American invasive species, *S. canadensis* and *S. gigantea* Aiton, can hybridize with the European native *S. virgaurea* L. producing the hybrids *S. × niederederi* Khek (hybrid between *S. canadensis* and *S. virgaurea*) and *S. × snarskisii* (hybrid between *S. gigantea* and *S. virgaurea*) [5,6,7,8,9]. In 2004, *S. canadensis*, together with 17 other plant species was recorded as an invasive weed in Lithuania [10]. Canadian goldenrod is considered to be one of the greatest threats to biodiversity and native species communities increasing the negative impact on natural ecosystems along with factors such as habitat degradation, change in landscape, pollution, exploitation and climate change [11,12].

*S. canadensis* contains a wide range of bioactive secondary metabolites, *viz.* flavonoids, saponins, polyacetylenes, (poly)phenolic acids, phenolic glycosides, terpenoids, etc. [3,13,14,15,16,17,18,19,20,21,22,23,24,25,26,27,28,29,30,31,32,33,34,35,36,37,38,39,40,41,42,43,44,45,46,47,48,49]. It is an essential-oil bearing plant. The chemical composition of the essential oils (EOs) and extracts of Canadian goldenrod has been relatively poorly studied. Previous studies have confirmed α-, *β*-pinene, sabinene, limonene, *β*-myrcene, *trans*-verbenol, bornyl acetate, thymol, germacrene D, various cadinene isomers, *β*-elemene, cyclocolorenone, caryophyllene oxide and 6-*epi*-*β*-cubebene, among major constituents in the EOs of fresh or dried *S. canadensis* inflorescences, leaves and stems [13,14,15,16,17,18,19,20,21,22,23,24,25,26,27,28,29,30]. A very limited number of publications are related to the Canadian goldenrod root EOs [31]. In the aforementioned study [31], it was shown that the root EO containing major compounds thymol, α-copaene and carvacrol, exhibited significant antibacterial activity against *Enterococcus faecalis* and *Escherichia coli* whereas it demonstrated moderate antifungal activity against *Candida albicans.*

Essential oils of aerial parts Canadian goldenrod exhibited cytotoxic [18,19,20], antiproliferative [29], antimicrobial [21,22,25] and insecticidal properties against *Culex quinquefasciatus*, *Spodoptera littoralis* and *Musca domestica* [28]. The vapor of the EOs of this plant effectively inhibits *Botrytis cinerea* growth and preserves the postharvest quality of strawberries [23]. Additionally, it was evaluated that EOs of *S. canadensis* had an impact on the germination of four weed species, i.e., *Amaranthus retroflexus* L., *Avena fatua* L., *Bromus secalinus* L. and *Centaurea cyanus* L.; influenced the germination of three crops, i.e., *Avena sativa* L., *Brassica napus* L. and *Zea mays* L. [25], and showed phytotoxic activity on radish (*Raphanus sativa* L.) and garden cress (*Lepidium sativum* L.) [24]. As we know, there are no studies to date on allelopathic activity of *S. canadensis* root EOs.

Several studies have focused on evaluation of the chemical composition and biological properties of various *S. canadensis* extracts [3,32,33,34,35,36,37,38,39,40,41,42,43,44,45,46,47,48,49]. The leaves and flowers of the plant contain a wide range of bioactive constituents that are responsible for its antioxidant, antimicrobial, antibacterial, antimutagenic, cytotoxic, anti-inflammatory, spasmolytic and diuretic properties [38,40,41,42,43,44,45,46]. Polyphenolic-polysaccharide-protein complex isolated from flowers of *S. canadensis* showed strong antitussive and anti-asthmatic activity [3].

Some studies were undertaken to elucidate the role of Canadian goldenrod allelopathy. The crude extracts from both underground and aerial parts of *S. canadensis* (growing in its native area in the USA and in an invaded area in China) showed allelopathic effects on seed germination of *Kummerowia striata* (Thunb.) Schindl., which is a native Chinese plant [50]. The crude extracts of roots of *S. canadensis* from five American native populations (from the east coast of the United States) and eight invasive European populations (from different sites in Austria, Czech Republic, France, Germany and Switzerland) were tested on the growth of seven competing native European plant species [51]. The allelopathic effects of *S. canadensis* (using leaf extracts) were evaluated on seed germination and growth of *Lactuca sativa* treated with five types of acid deposition using different sulfuric and nitric acid ratio [52]. Three types of aqueous extracts (decoction, infusion, and macerate) from the Canadian goldenrod leaves were used to examine the germination and early stages of development of *Trifolium pratense* L. and *Raphanus sativus* L. var. *radicula* Pers.; all the extracts had a negative influence on germination [53,54]. The allelopathic effects of various extracts from *S. canadensis* against seed germination and seedling growth of some plants, such as mulberry (*Morus alba* L.), morning glory (*Pharbitis nil* (L.) Roth), wheat (*Triticum aestivum* L.), setaria (*Setaria viridis* (L.) Beauv.), rape (*Brassica campestris* L.), indica rice (*Oryza sativa* L.), oilseed rape (*Brassica napus* L.), *B. campestris* subsp. *chinensis* (L.) Makino [*B. chinensis*], tomato (*Lycopersicon esculentum* L.), false daisy (*Eclipta prostrata* L.), lettuce (*Lactuca sativa* L.), radish (*Raphanus sativus* var. *radicula* Per), soybean (*Glycine max* (L.) Merr.) and barley (*Hordeum vulgare* L.); and on the rhizogenesis processes of freshly cut chrysanthemums (*Chrysanthemum × koreanum* Makai) shoots were assessed [49,55,56,57,58].

Additionally, several research studies have focused on the autoallelopathic effect of *S. canadensis* extracts [59,60]. The aqueous extracts prepared from various parts of Canadian goldenrod and giant goldenrod were tested on both goldenrods [59]. The most effective were extracts from rhizomes against the growth and development of Canadian goldenrod and giant goldenrod, which caused a reduction of fresh biomass of goldenrods up to 42%, compared to water-treated control in the above study. Another paper verified allelopathic and autotoxic effects of aqueous leaf extracts of *S. virgaurea*, *S. canadensis*, *S. gigantea* and *S. × niederederi* on two congeneric pairs of species *Festuca pratensis* Huds. and *F. rubra* L., and *Solidago* occurring naturally in communities with the tested *Solidago* species [60]. Germination and seedling growth of *F. rubra* were inhibited by all *Solidago* extracts more than were those characteristics of *F. pratensis*, while *S. canadensis* was more sensitive to its own and congeneric extracts than was *S. × niederederi* [60].

It must be mentioned that the allelopathic effect of *S. canadensis* on other plants has not been studied sufficiently [49,50,51,52,53,54,55,56,57,58]. To the best of our knowledge, there are very few studies on the allelopathy of Canadian goldenrod EOs, especially root oils. The aim of the present study is to evaluate allelopathic properties of various extracts and EOs obtained from different parts (inflorescences, leaves and roots) of invasive *S. canadensis* on the seed germination and growth of lettuce (*Lactuca sativa* L.) and garden pepper cress (*Lepidium sativum* L.); and compare this activity with other *Solidago* species *S. virgaurea* (native species in Lithuania) and hybrid *S. × niederederi* Khek (*S. canadensis* and *S. virgaurea*) collected from the same growing locality.

## 2. Results

### 2.1. Soil Characteristics (Conductivity, pH and Major Elements)

Soil parameters, such as conductivity and pH of *Solidago* species (*S. canadensis, S. virgaurea* and *S. × niederederi*) growing locality presented in Table 1. The data of the elemental analysis of the soil is shown in Table 2.

### 2.2. Chemical Composition of Solidago canadensis Methanol/Water Extracts

Twenty-two compounds were identified tentatively in the leaf, inflorescence and root methanol/water (70:30 *v/v*) extracts of Canadian goldenrod (Table 3). All constituents were detected by DAD and TOF in positive or negative ionization mode. Some compounds provided m/z ions by both (positive and negative) ionizations. 

### 2.3. Chemical Composition of Volatile Organic Compounds (VOCs) in Solidago canadensis Water/Diethyl Ether Extracts

The main compositional data of chemical analysis performed by GC/FID and GC/MS of *S. canadensis* extracts of different acidity obtained from various plant organs are presented in Table 4.

### 2.4. Chemical Composition of Solidago canadensis Essential Oils (EOs)

A gas chromatography (GC) analysis equipped with FID and GC/MS techniques (respectively for quantitative and qualitative purposes) was applied for chemical analysis of *S. canadensis* EOs obtained from various plant parts such as inflorescences, leaves and roots. The principal compositional data are presented in Table 5. In total, up to 98 compounds were identified in the EOs, comprising up to 95.4%.

### 2.5. Allelopathic Effects of Solidago canadensis Water/Diethyl Ether Extracts

**Figure 1:**
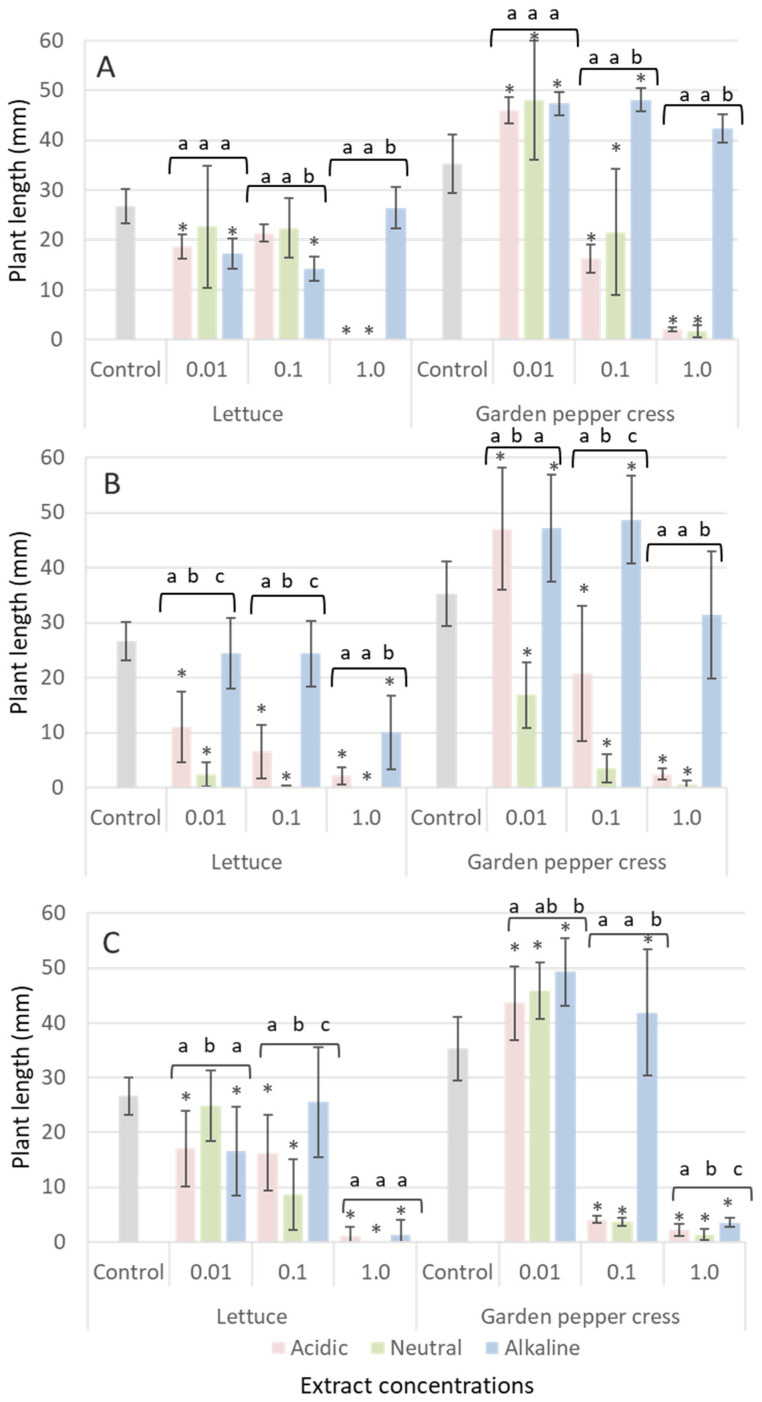
Inhibitory effect of *S. canadensis* water/diethyl ether extracts (acidic, neutral and alkaline fractions at 0.01, 0.1 and 1.0 relative concentration ranges) of roots (**A**), leaves (**B**) and inflorescence (**C**) on germination and growth (mm) of lettuce (*Lactuca sativa*) and garden pepper cress (*Lepidium sativum*) (one-way ANOVA test, df = 9, *p* < 0.05). Data are presented as means of treatments (n = 60) ± SD (bars); asterix (*) indicates statistically significant difference compared to the control; letters (a, b and c) denote statistically significant difference between fractions (Tukey’s test, df = 2, *p* < 0.05).

The allelopathic effect of leaf, inflorescence and root etheric extracts of *S. canadensis* on seed germination and growth of *Lactuca sativa* and *Lepidium sativum* is presented in Figure 1.

### 2.6. Relative Germination (RG) and Vigor Index (VI) of Lettuce (Lactuca sativa L.) and Garden Pepper Cress (Lepidium sativum L.) Affected by S. canadensis, S. virgaurea and S. × niederederi Extracts

**Figure 2:**
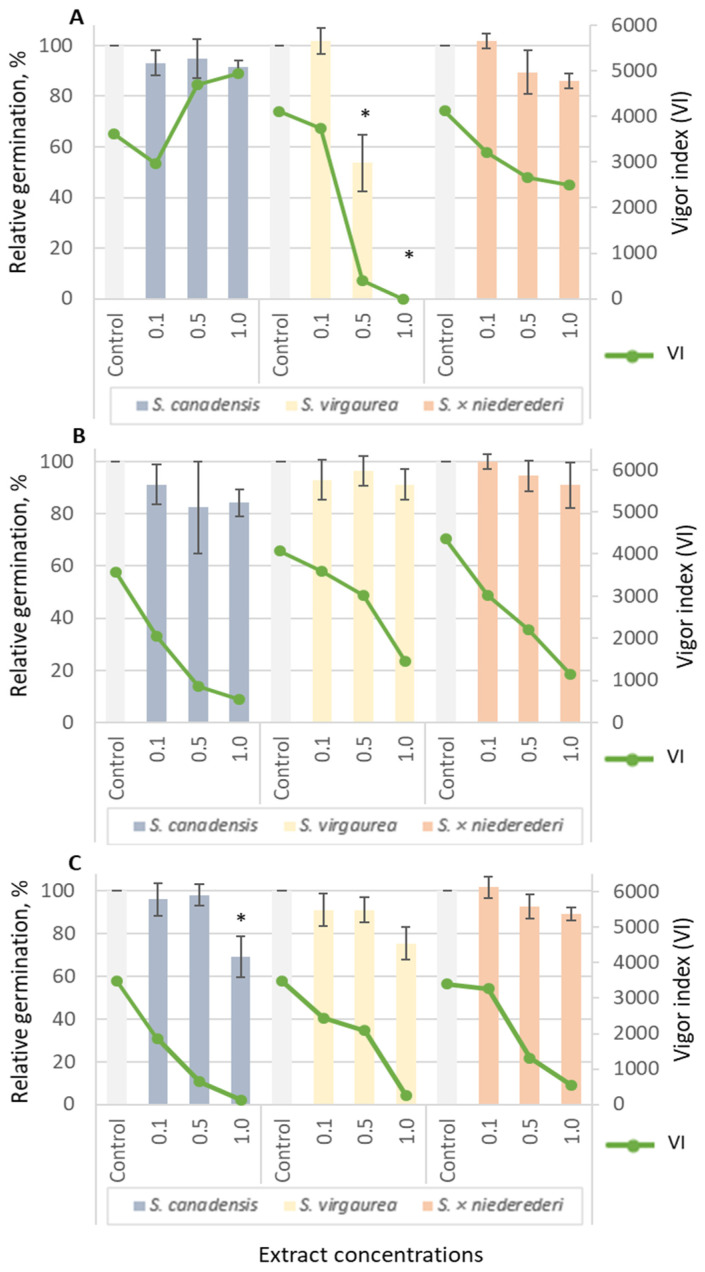
Inhibitory effect of aqueous extracts from *S. canadensis*, *S. virgaurea* and *S. × niederederi* roots (**A**), leaves (**B**) and inflorescences (**C**) on relative germination (RG) and vigor index (VI) of lettuce seeds (G test, *p* < 0.05). Data are presented as the mean of treatments (n = 60) ± SD (bars); asterisk (*) indicates a statistically significant difference compared to the control samples.

**Figure 3:**
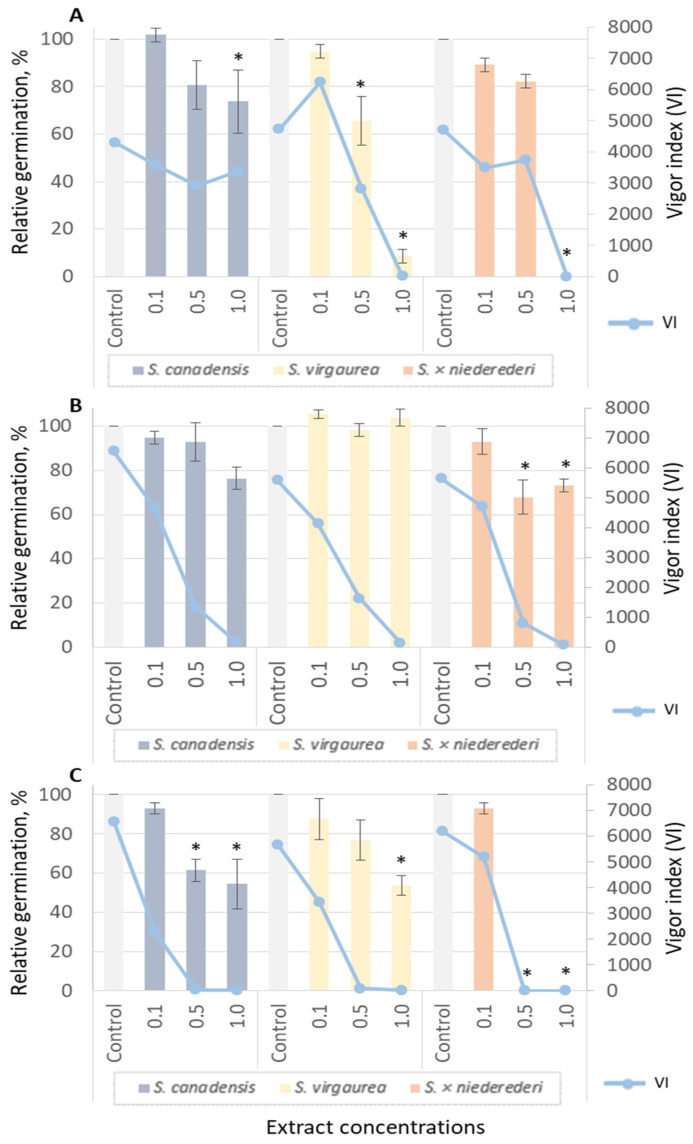
Effect of aqueous extracts from *S. canadensis*, *S. virgaurea* and *S. × niederederi* roots (**A**), leaves (**B**) and inflorescences (**C**) on relative germination (RG) and vigor index (VI) of garden pepper cress seeds (G test, *p* < 0.05). Data are presented as the mean of treatments (n = 60) ± SD (bars); asterisk (*) indicates a statistically significant difference compared to the control.

Extracts of *S. canadensis*, *S. virgaurea* and *S. × niederederi* Khek (hybrid between *S. canadensis* and *S. virgaurea*) significantly affected seed germination and growth of tested lettuce (*Lactuca sativa*) (Figure 2) and garden pepper cress (*Lepidium sativum*) (Figure 3). The root extracts of *S. virgaurea* showed the highest effect on lettuce and pepper cress’s relative germination (RG) and vigor index (VI).

Data of inhibitory effects of root, leaf and inflorescence aqueous extracts of *S. canadensis*, *S. virgaurea* and *S. × niederederi* on germination rate (GR), relative germination (RG) and vigor index (VI) of lettuce and garden pepper cress seeds are presented in Tables S2–S4.

### 2.7. Allelopathic Effects of Water Extracts of Various Solidago Species: S. canadensis, S. virgaurea and S. × niederederi

**Figure 4:**
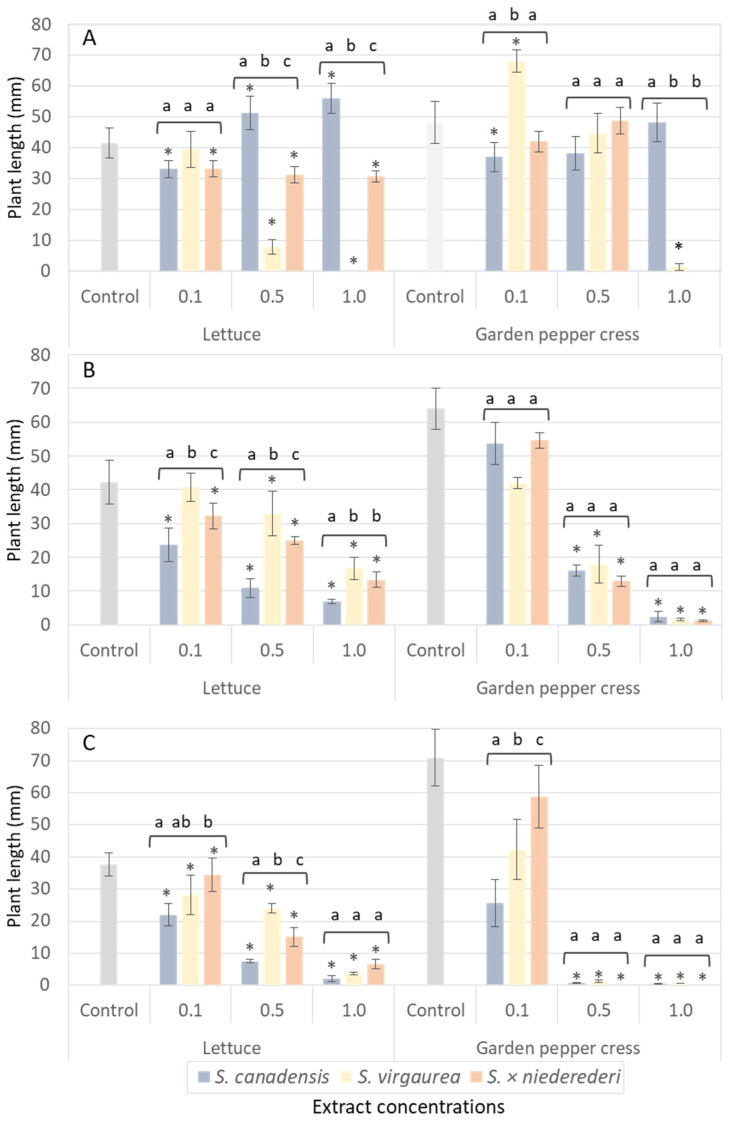
Effect of aqueous extracts from roots (**A**), leaves (**B**) and inflorescences (**C**) of *S. canadensis*, *S. virgaurea* and *S. × niederederi* on the seed germination and growth of lettuce and garden pepper cress at 0.1, 0.5 and 1.0 relative concentration (one-way ANOVA test, df = 3, *p* < 0.05). Data are presented as mean of treatments (n = 60) ± SD (bars); asterix (*) indicates statistically significant differences compared to the control; letters (a, b and c) denote statistically significant differences between various species (Tukey’s test, *p* < 0.05).

The allelopathic effect of leaf, inflorescence and root aqueous extracts of *S. canadensis*, *S. virgaurea* and *S. × niederederi* on the seed germination and growth of *Lactuca sativa* and *Lepidium sativum* is presented in Figure 4.

### 2.8. Inhibitory Effect of Solidago canadensis EOs on Seed Germination and Seedling Growth of Lettuce (Lactuca sativa) and Garden Pepper Cress (Lepidium sativum)

**Figure 5:**
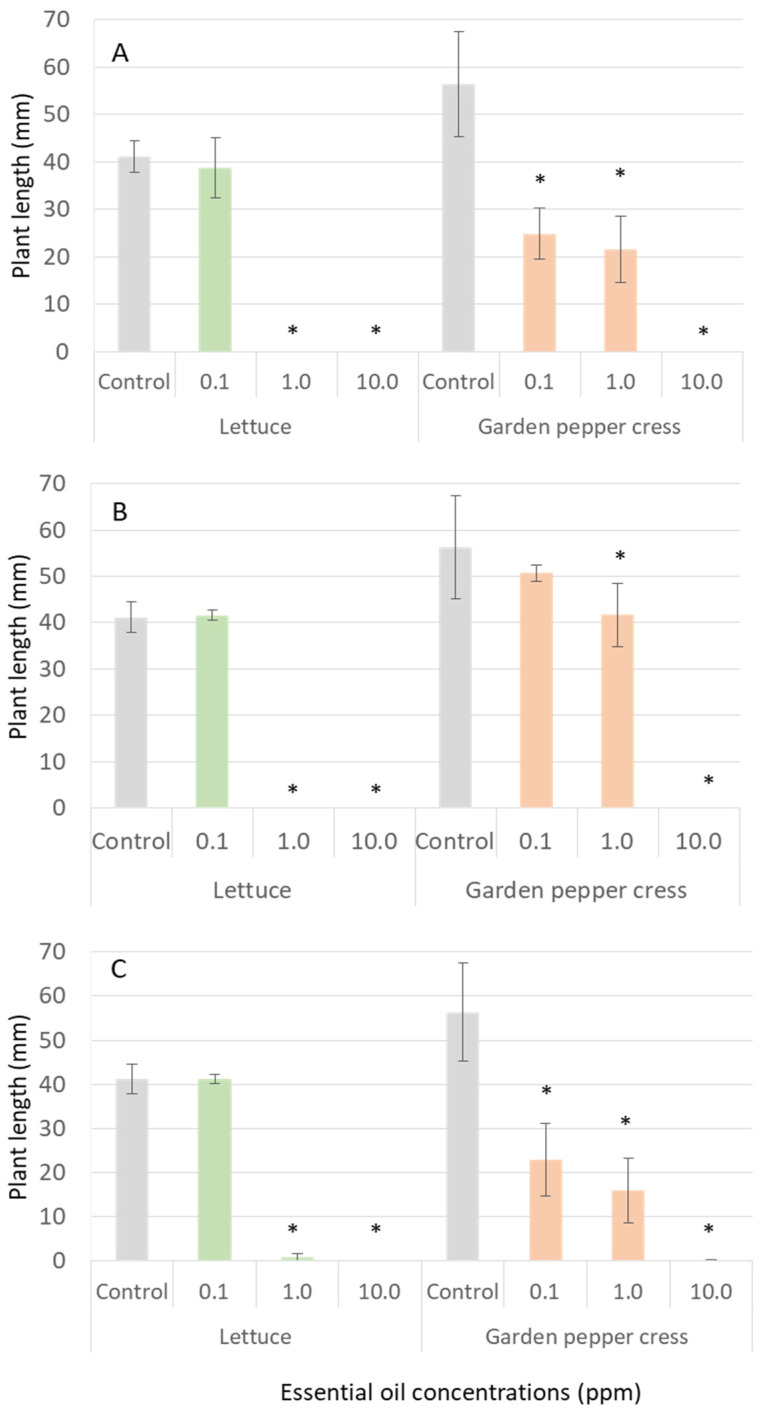
Inhibitory effect of *S. canadensis* essential oils (roots—(**A**), leaves—(**B**) and inflorescences—(**C**)) on germination and growth of lettuce and garden pepper cress (one-way ANOVA test, df = 3, *p* < 0.05). Data are presented as the mean of treatments (n = 60) ± SD (bars); asterisk (*) indicates a statistically significant difference compared to the control.

The essential oils of *S. canadensis* significantly affected seed germination and growth of tested lettuce and garden pepper cress (Figure 5). The EOs of Canadian goldenrod roots and inflorescences showed the highest effect on lettuce and garden pepper cress growth (Figure 5A,C).

## 3. Discussion

It is known that *S. canadensis* L., being an expansive perennial weed, forms persistent, species-poor plant communities. According to the European habitat classification system (EUNIS), the growing sites (Lithuania, Vilnius, Trakai municipality, Lentvaris) of *S. canadensis*, *S. virgaurea* and hybrid *S. × niederederi* plants can be attributed to the category of anthropogenic herb stands with dry perennial anthropogenic herbaceous vegetation (EUNIS2020 code V38 and E5.1) [62]. Diagnostic species of the growing locality was mugwort (*Artemisia vulgaris* L.); and characteristic species are the following: couch grass (*Elytrigia repens* (L.) Gould), mugwort (*Artemisia vulgaris* L.), common yarrow (*Achillea millefolium* L.), field bindweed (*Convolvulus arvensis* L.), cat grass (*Dactylis glomerata* L.), meadow-grass (*Poa pratensis* L.), creeping thistle (*Cirsium arvense* (L.) Scop), common nettle (*Urtica dioica* L.), narrowleaf plantain (*Plantago lanceolata* L.), wild carrot (*Daucus carota* L.), common dandelion (*Taraxacum* sect. *Taraxacum* F.H.Wig.), Canadian horseweed (*Erigeron canadensis* L.), blueweed (*Echium vulgare* L.), hop clover (*Medicago lupulina* L.), common chicory (*Cichorium intybus* L.), common tansy (*Tanacetum vulgare* L.), wood small-reed (*Calamagrostis epigejos* (L.) Roth ), white campion (*Silene latifolia* Poir.), St. John’s wort (*Hypericum perforatum* L.) and false mayweed (*Tripleurospermum maritimum* (L.) W.D.J. Koch) in this habitat.

Around 50% of this territory was occupied by *S. canadensis* plants, distributed almost evenly over the area (Figure S1 in Supplementary Materials). The rapid spread of the herb is due to several reasons. Canadian goldenrod plant has broad climatic tolerance; also it is tolerant to a wide range of soil nature and fertility, texture conditions and soil pH [63,64,65]. Another important aspect is plant lifecycle and dispersion. *S. canadensis* is a perennial rhizomatous hemicryptophyte and spreads both via seeds and underground rhizomes. The plant has a relatively long flowering time (from late July to mid-September or October). Individual clones are long-lived (can reach up to 100 years) and spread fast with each year. One sprout of Canadian goldenrod can mature up to 20 000 seeds per season. Seeds are responsible for long-distance dispersal and the colonization of unoccupied sites. However, rhizomatous growth is significant for the expansion of *S. canadensis* populations.

The soil of the growing habitat was investigated and characterized by some main parameters. Soil pH values ranged from 6.15 ± 0.07 to 6.65 ± 0.27 and conductivity was from 92.33 ± 0.55 to 125.63 ± 6.04 µS/cm (Table 1). According to the soil map of the National Atlas of Lithuania (the new FAO classification of Lithuanian soils (LTDK-99) has been adopted), typical alluvial soils have been found in this area. The elemental composition of the soil was typical (Table 2) and concentrations of hazardous and heavy metals were below limitary values according to the Lithuanian health regulations (V-114 HN60:2004) [66].

The success of the Canadian goldenrod invasion may be explained by its allelopathic compounds. It is known that allelochemicals can be obtained from different parts of the plant (leaves, stems, flowers, buds, fruits and roots) and can affect seed germination and root formation on neighboring plants, as well as the growth of the whole plant. The allelopathic effect of *S. canadensis* on other plants has not been studied very widely. Thus, it is important to identify allelopathic compounds and their role in the spread of invasive species. Mainly polyphenolic acids and flavonoids were identified in the methanol/water extracts from *S. canadensis* inflorescences, leaves and roots (Table 3). The identity of some compounds was confirmed by matching of chromatographic data with corresponding reference compounds. Most of the determined constituents were identified formerly in Canadian goldenrod extracts [32,35,36,46,47,48,49]. The retention times of compounds varied slightly for each sample. Three compounds with a molecular mass of 354 were identified by both positive and negative ionization and by DAD as neochlorogenic, chlorogenic and 4-*O*-caffeoylquinic acid in Canadian goldenrod leaf and inflorescence extracts. Feruloylquinic acid and narcissin were found only in the leaf extract, while caffeoylshikimic acid and its glucoside, cinnamic and ferulic acids were determined purely in the root extract. In addition, two saponins and cinnamtannin A2 were identified in the Canadian goldenrod root extract. No one constituent was determined in extracts from all plant parts. It should be mentioned that some common compounds such as afzelin, astragalin, kaempferol, nicotiflorin, quercetin, quercitrin and some others previously identified in *S. canadensis* extracts [32,35,36,46,48,49] were determined in the present study in small amounts or even under detection limits.

The compositional data of VOCs in Canadian goldenrod extracts prepared according to the method described in Chapter 4.3.2 are presented in Table 4. The percentage of individual compounds varied slightly depending on the pH of solutions. Leaf extracts contained major compounds *trans*-verbenol (≤24.8%), verbenone (≤9.4%) and *trans*-pinocarveol (≤6.0%). The main volatile composition of *S. canadensis* inflorescence water/diethyl ether extract was the following: eugenol (≤16.1%), 4-vinyl-guaiacol (≤11.5%) and 7,8-dihydro-3-oxo-*α*-ionol (≤8.7%). Root extracts were characterized by the main constituents α-terpineol (≤23.3%), 1,8-cineole (≤16.1%) and *α*-pinene (≤10.2%).

α-Pinene (21.6%), germacrene D (15.1%), limonene (10.2%) and lupenyl acetate (9.8%) were found to be major constituents in the inflorescence EOs of *S. canadensis* (Table 5). These compounds were determined as principal constituents in previous studies of Canadian golden EOs [14,17,19,26,27,28,29,30]. The investigated leaf EOs were characterized by *trans*-verbenol (21.3%) and verbenone (12.5%). The following compounds are not as commonly found in Canadian goldenrod EOs. To date, the number of studies on *S. canadensis* root EOs is very limited. The investigated oil, containing limonene (35.0%) and *β*-pinene (26.2%) as predominant constituents differed drastically from the root EO obtained by steam distillation and comprised the major compounds thymol (20.3%), α-copaene (6.3%) and carvacrol (5.5%) of Indian origin (Bhimtal) [31].

The inhibitory effects of *S. canadensis* leaf, inflorescence and root exudates on seed germination and growth of lettuce (*Lactuca sativa*) and garden pepper cress (*Lepidium sativum*) differed slightly. Canadian goldenrod leaf (Figure 1B) and inflorescence (Figure 1C) extracts were found to be the most active and seed germination of lettuce and garden pepper cress was inhibited up to 100% at a relative concentration of 1.0. However, the neutral leaf extract showed the highest inhibitory effect in all relative concentrations (1.0; 0.1; 0.01) suppressing growth of *Lactuca sativa* (from 0 to 2.3 mm compared with 22.7 mm for control samples) and *Lepidium sativum* (from 0.5 to 16.8 mm compared with 35.3 mm in the control group) (Figure 1).

For comparison, different extracts of various *Solidago* species were tested in order to evaluate allelopathic properties. *Solidago canadensis*, *S. virgaurea* and *S. × niederederi* extracts significantly affected seed germination and growth of tested lettuce (Figure 2) and garden pepper cress (Figure 3). *Solidago virgaurea* root extracts have shown the highest effect on lettuce and pepper cress relative germination (RG) and vigor index (VI). At a relative concentration of 0.5, the RG of lettuce reached 53.57% (Figure 2A); pepper cress (65.52%) (Figure 3A) (G test, *p* < 0.05) and VI were 390.83 and 2829.79, respectively. At the highest tested concentration (1.0), germination RG was 0% for lettuce and 8.62% for pepper cress. Aqueous root extracts of *S. × niederederi* significantly affected only garden pepper cress RG at 1.0 concentration and seed germination was totally suppressed (Figure 3, A). Leaf extracts from *S. × niederederi* had an effect only on pepper cress’s RG and were 67.86% and 73.21% at 0.5 and 1.0 relative concentrations (G test, *p* < 0.05) (Figure 3B). *Solidago canadensis* and *S. × niederederi* inflorescence extracts affected lettuce (Figure 2C) and pepper cress’s RG and VI (Figure 3C). However, *S. × niederederi* inflorescence extract (at relative concentrations 0.5 and 1.0) suppressed garden pepper cress seed RG completely (Figure 3C) (G test, *p* < 0.05).

For comparative purposes, aqueous extracts of native and invasive *Solidago* species growing at the same locality were tested under laboratory conditions (Figure 4). *Solidago canadensis*, *S. virgaurea* and *S. × niederederi* extracts significantly affected the seed germination and growth of lettuce (*Lactuca sativa*) and garden pepper cress (*Lepidium sativum*). It is evident that *Solidago*’s inhibitory effects differed on species. In the case of *S. virgaurea,* root and inflorescence extracts indicated the highest allelopathic activity, while for *S. canadensis* and *S. × niederederi* both leaf and flower extracts demonstrated similar inhibitory effects on model plants (Figure 4). The strongest inhibitory effects were caused by aqueous extracts of inflorescences of all tested *Solidago* species at 1.0 and 0.5 of relative concentration. In this case, seed germination and growth was reduced from 81.2 to 94.8% for lettuce (*L. sativa);* and 100% for garden pepper cress (*L. sativum*).

The allelopathic effects of the plant extracts are implemented usually by using the whole plant or leaves only [49,50,51,52,53,54,55,56,57,58]. Contrary to this, our research confirmed the importance of studying individual morphological parts (leaves, flowering tops and roots) and the effects of their aqueous/etheric extracts. To the best of our knowledge, allelopathy of *Solidago* spp. root extracts were investigated for the first time. Aqueous extract of *S. canadensis, S. virgaurea* and *S. × niederederi* inflorescences showed the highest inhibitory effect on seed germination and growth of model plants (*Lactuca sativa* and *Lepidium sativum*). In comparison to parental plant species hybrid (*S. × niederederi*) also effectively suppressed germination and growth of the tested plants. Comparing model plants, it was noticed that garden pepper cress (*Lepidium sativum*) was more susceptible to *Solidago* sp. inhibitory effects than lettuce (*Lactuca sativa*).

A comparison of our results on allelopathy of *Solidago* spp. extracts with previously published data [49,50,51,52,53,54,55,56,57,58] is complicated for several reasons: different preparation of extracts, their chemical composition is not always known and various model plant species have been used for the tests.

The experimental data evaluated that *S. canadensis* EOs significantly affected seed germination and growth of lettuce (*Lactuca sativa*) and garden pepper cress (*Lepidium sativum*) (Figure 5). Root EOs, comprised mainly of limonene (35.0%) and *β*-pinene (26.2%) and inflorescence oils, containing main components α-pinene (21.6%), germacrene D (15.1%), limonene (10.2%) and lupenyl acetate (9.8%) exhibited the highest inhibitory effect on lettuce and garden pepper cress growth (Figure 5A). Root EOs at the highest concentrations (1.0 and 10 ppm) suppressed lettuce seed germination completely. Garden pepper cress seed growth was affected at all concentrations and plant lengths were from 24.85 ± 5.36 mm (at an EO concentration 0.1 ppm) to 21.63 ± 6.98 mm (at EO 1.0 ppm), and 0 mm (at EO 10 ppm) in comparison to 56.33 ± 8.08 mm for the control samples. Inflorescence EOs at the highest concentrations (1.0 and 10 ppm) suppressed lettuce seed germination and growth almost completely (plant lengths from 0.85 ± 0.81 mm to 0 mm, respectively) (Figure 5C). Garden pepper cress seeds growth was affected at all concentrations and plant lengths were from 22.97 ± 8.20 mm (at EO concentration 0.1 ppm) to 15.98 ± 7.35 mm (at EO 1.0 ppm), and 0.15 mm ± 0.13 mm (at EO 10 ppm) in comparison to 56.33 ± 8.08 mm for the control samples. From this experiment, we can state that root and inflorescence EOs showed the highest inhibitory effects on model plant seed germination and growth. It is noticed that lettuce seeds were more susceptible to *S. canadensis* EO’s inhibitory effects than garden pepper cress seeds. Our data are in correlation with allelopathy of EOs of aerial parts of *S. canadensis* collected from natural populations in Slovakia [24] and Poland [25]. The oils of different chemical composition (containing *α*- or *β*-pinene, germacrene D, limonene, thymol, *epi*-bicyclosesquiphellandrene, *β*-cadinene, *γ*-cadinene, *δ*-cadinene, *α*-or *γ*-muurolene, *α*-cubebene or *β*-elemene as main components) significantly inhibited seed germination and growth of garden pepper cress and other model plant species [24,25].

## 4. Materials and Methods

### 4.1. Soil Analysis

Preparation of soil samples for elemental analysis, conductivity and pH measurements was done in such way: all samples of the soil were dried at room temperature, later ground and sieved through a bolt (2.0–2.5 mm of perforation).

A mixture of 20 mL of sifted soil and 40 mL of deionized water was rested for 1 h in an ultrasonic bath; later the mixture was filtered and the conductivity of aliquots was measured using the conductivity and temperature meter AD3000 EC/TDC (Adwa, Szeged, Hungary).

Sample preparation for pH measurements was as follows: a mixture of 5 mL of soil and 25 mL of deionized water was shaken for 1 h in an ultrasonic bath, stored for 2 h and filtered; measurements of the aliquots were performed by pH-meter Orion 3 Star (Thermo Fisher Scientific, Waltham, MA, USA), calibrated using buffer solutions of pH 4.01, 7.00 and 10.04.

Elemental analyses were undertaken with the inductively coupled plasma–optical emission spectroscopy (ICP-OES) method. Procedure of sample preparation: 5 g of soil and 50 mL of 1 M HCl was stirred for 30 s, and then rested for 24 h. The mixtures were filtered and analysis was performed using the Optima 700 DV spectrometer (Perkin Elmer, Waltham, MA, USA).

### 4.2. Plant Material

**Figure 6:**
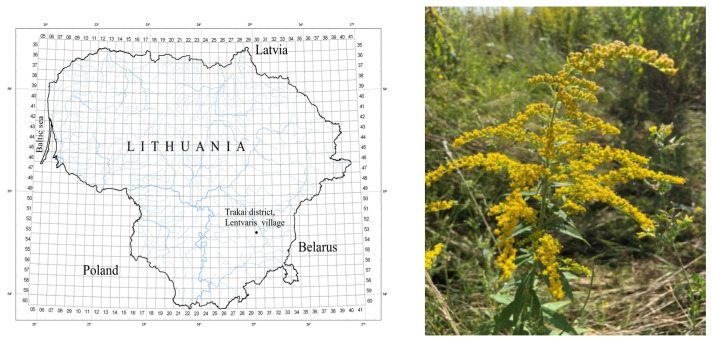
Geographical indication of sampling site in Eastern Lithuania (Vilnius, Trakai municipality, Lentvaris). Photo of *S. canadensis* (courtesy D. Stancelytė) from the investigated population.

*Solidago canadensis*, *S. virgaurea* and *S. × niederederi* plants (up to 2.5–3.0 kg) were collected at full flowering stage (in August 2020 and 2021) in a derelict field (Eastern Lithuania, Vilnius, Trakai municipality, Lentvaris, 54°38′53.5° N 25°07′37.9° E). The habitat is depicted on the geographic information system map (Figure 6). The area of the investigated populations was up to 150 m^2^. Plants were collected randomly from four different sampling sites at full flowering stage. Raw material (above- and below-ground plant parts) was taken immediately to the laboratory and dried at room temperature (20–25 °C) under shade conditions for 2 weeks. Leaves, inflorescences and roots were separated before drying.

Plant material was identified by Dr. M. Rasimavičius, and voucher specimens of *S. canadensis, S. virgaurea* and *S. × niederederi* were deposited at the Vilnius University Herbarium (WI, Vilnius, Lithuania) with code numbers P33666, P33663 and P33664, respectively.

*Lactuca sativa* and *Lepidium sativum* seeds were selected as the model plants. Seeds were bought from a local vegetable market.

### 4.3. Preparation of Various Plant Extracts

#### 4.3.1. Essential Oil Isolation

The essential oils from *S. canadensis* were isolated by hydrodistillation of dried material (up to 100 g each) in a Clevenger-type apparatus for 2 h according to the European Pharmacopoeia. The ratio of plant material to water was 1:20. A yellow-gray, greasy mass with a sweet characteristic odor was obtained. Hydrodistillation yielded 1.25, 1.11 and 0.95% (*v/w*, on a dry weight basis) of EO from *S. canadensis* inflorescences, leaves and roots, respectively. Yields of the EOs slightly ranged according to the collection time (2020 and 2021). The obtained oils were dried over anhydrous sodium sulfate, kept in closed dark vials and stored in a refrigerator; the samples were diluted with a mixture of pentane and diethyl ether (1:1) before analysis.

#### 4.3.2. Preparation of *S. canadensis*, *S. virgaurea*, *S. × niederederi* Extracts for Allelopathic Tests

Thirty g of crushed herbal material (separately: flowers, leaves and roots) and 500 mL of distilled water were kept at 40 °C for 24 h, then filtered and three portions of 25 mL were prepared. In order to obtain acidic (pH ca. 3) and alkaline (pH ca. 11) solutions, 0.1 M HCl and 0.5 M KOH were applied. An amount of 1.5 mL of the solution used for allelopathic tests was considered as 1.0 of relative concentration.

Then, each portion was extracted with 5 mL of diethyl ether for 5 min. Diethyl ether was evaporated. The dry residues were dissolved in deionized water before germination assay.

#### 4.3.3. Preparation of *S. canadensis* Extracts for HPLC-DAD-TOF Analysis

Samples of air-dried flowers, leaves and roots of *S. canadensis* were ground into a homogenous powder and protected from light and humidity until analysis. Up to 1 g of crushed herbal material and 15 mL of solvent (mixture of methanol and water (70:30, *v/v*)) were used for extraction. The extraction procedure was performed in an ultrasonic bath at room temperature for 50 min. The mixture was filtered through a filter paper for qualitative analysis (pore size 11 µm) using nylon syringe filters (0.22 mm).

### 4.4. Gas Chromatographic Analysis of Solidago canadensis EOs and Extracts (Water/Diethyl Ether)

#### 4.4.1. GC (Flame-Ionization Detector FID) Analysis

Quantitative analyses of the EOs and extracts were carried out on HP 5890II chromatograph equipped with an FID (Hewlett Packard, Palo Alto, CA, USA), using DB-5 ((5%-phenyl)-methylpolysiloxane; 50 m × 0.32 mm × 0.25 μm) and HP-FFAP (polyethylene glycol 30 m × 0.25 mm i.d., film thickness 0.25 μm) capillary columns (Agilent, J&W Scientific, Santa Clara, CA, USA). The GC oven temperature was programmed as follows: from 50 °C (isothermal for 1 min) increased to 160 °C (isothermal for 2 min) at a rate of 5 °C/min, then increased to 250 °C at a rate of 10 °C/min, and the final temperature was kept for 4 min. The temperature of the injector and detector was maintained at 250 °C. The flow rate of carrier gas (hydrogen) was 1 mL/min. At least 3 replicates per analysis were performed.

#### 4.4.2. GC-MS Analysis

Analyses were performed on a chromatograph Shimadzu GC-2010 PLUS (Shimadzu, Kyoto, Japan) interfaced to a Shimadzu GC-MS-QP2010 ULTRA mass spectrometer (Shimadzu, Kyoto, Japan) and fitted with a capillary column Rxi-5 MS (Restek, Bellefonte, PA, USA), (5%-phenyl)-methylpolysiloxane 33 m × 0.25 mm i.d., film thickness 0.25 µm).

The conditions of chromatographic separation were the same as for GC (FID) analysis. The temperature of the injector and detector was 250 °C. The flow rate of carrier gas (helium) was 1 mL/min, split 1:20. At least 2 replicates per analysis were performed. The temperature of ion source was 220 °C. Mass spectra in electron mode were generated at 70 eV, 0.97 scans/second, mass range 33–400 *m*/*z*.

#### 4.4.3. Identification of Individual Components

The percentage composition of the EOs and VOCs was computed from GC peak areas without correction factors. Qualitative analysis was based on comparison of retention indexes on both columns (polar and nonpolar), co-injection of some reference terpenoids (*α*-, *β*-pinene, 1,8-cineole, linalool, camphor, *β*-caryophyllene, *α*-humulene and caryophyllene oxide) and C_8_–C_28_ n-alkane series; and mass spectra with corresponding data in the literature [61] and computer mass spectra libraries (Flavors and Fragrance of Natural and Synthetic Compounds 2 (FFNSC 2), Wiley and NIST). Identification was approved when computer matching with the mass spectral libraries showed a probability above 90%. The relative proportions of the oils and extracts constituents were expressed as percentages obtained by peak area normalization, all relative-response factors being taken as one.

### 4.5. HPLC-DAD-MS (TOF) Analysis Solidago canadensis Extracts

Methanol/water (70:30, *v/v*) extracts from inflorescences, leaves and roots were analyzed by an HPLC technique using a system HPLC/Diode Array Detector (DAD)/Time of Flight (TOF) (Agilent 1260 Infinity (Agilent Technologies, Waldbronn, Germany) and Agilent 6224 TOF (Agilent Technologies, Santa Clara, CA, USA) equipped with a reverse phase column ZORBAX Eclipse XDB (C18, 5 μm particle size, 150 × 4.6 mm, Agilent Technologies, Santa Clara, CA, USA). The column temperature was maintained at 25 °C. A gradient system was applied: A (deionized water, containing 0.1% of formic acid) and B (acetonitrile, containing 0.1% of formic acid). Chromatographic separation was performed at a flow rate of 0.8 mL/min in the HPLC system by the following stepwise gradient elution method: initial 95% (A)/5% (B); from 0 to 25 min from initial ratio to 0% (A)/100% (B); from 25 to 30 min: isocratic mode at 0% (A)/100% (B), from 30 to 35 min; from 0% (A)/100% (B) to 95% (A)/5% (B) and from 35 to 38 min isocratic mode at 95% (A)/5% (B). Ionization was performed by electrospray ionization interface (ESI) in positive and negative mode. Sample volume from 4 to 10 μL was injected by auto-sampler.

The MS (TOF) acquisition parameters were as follows: mass range 100–1700 m/z, rate 1.42 spectra/s, time 704.2 ms/spectrum. Ionization source conditions were drying gas temperature 300 °C, drying gas flow rate 3 L/min, nebulizer 15 psig, fragmentor voltage 125 V, skimmer 65 V. To ensure the mass accuracy of recorded data, continuous internal calibration with reference masses *m*/*z*: 121.050873, 149.02332, 322.048121, 922.009798, 1221.990637 and 1521.971475 (as per instrument standards, ref. nebulizer 5 psig) was performed.

### 4.6. Allelopathy of Solidago canadensis EOs

Allelopathic activity of *S. canadensis* EOs (from roots, leaves and inflorescences, separately) was tested in vivo, using model plant seeds (*Lactuca sativa* and *Lepidium sativum*). Different concentrations of *S. canadensis* EOs dissolved in dimethyl sulfoxide (DMSO) were added. Control tests were performed with DMSO in deionized water (1 μL/mL).

### 4.7. Bioassay for Seed Germination

*Lactuca sativa* and *Lepidium sativum* seeds were surface sterilized (by 70% ethanol for approximately 2 min) and then thoroughly washed thrice with deionized water. The seeds (20 per dish) were maintained in the dark at 25 °C in glass Petri dishes (Ø 9 cm) containing a sheet of Whatman filter paper and 1.5 mL of test or control (DMSO 1 μL/mL in deionized water) solution for 5 days. Three replicates were performed for each concentration. The number of germinated seeds was counted after 5 days of incubation time, and each seed was considered to have germinated when the radicle emerged (Figure S2 in Supplementary Materials). The plant root length and seedling height were measured with a caliper (Mitutoyo, Aurora, IL, USA) for comparisons among treatments.

### 4.8. Seedling Root Length (RL), Seedling Height (H), Germination Rate (GR), Relative Germination (RG), and Vigor Index (VI) Measurements

Twenty seedlings per Petri dish were measured after 5 days of incubation. Seedling root length (RL) and seedling height (H) were measured and are presented as plant length (PL). Germination rate (GR), relative germination (RG) and vigor index (VI) of *L. sativa* and *L. sativum* were determined according to the literature [67] (Table 6).

### 4.9. Statistical Data Analysis

The obtained results were statistically processed by calculating the Pearson correlation coefficient (*r*); the results were expressed as mean values, range intervals, and standard deviation (SD) values, using XLSTAT (trial version, Addinsoft 2014, Paris, France).

The PAST version 4.03 was applied for data statistical analysis, to determine reliability and significant differences. The comparison of independent groups was performed by one-way ANOVA test. Differences between the control and treatment groups were compared using Tukey test, applying a significance level of α = 0.05. The G test was used to determine statistically significant differences in the assessment of germination rate and relative germination. Statistically significant differences were set at *p* values equal to or lower than 0.05.

## 5. Conclusions

The ecosystem of invasive *Solidago canadensis* native to Lithuania and *Solidago virgaurea* and their hybrid *S. × niederederi* was investigated. Soil parameters and population characteristics of investigated area where three *Solidago* species grow collectively are defined in detail.

Allelopathic effects of the plant aqueous extract are usually implemented by using the whole plant or leaves only. However, our research confirmed the importance of studying individual morphological parts and the effects of their extracts and EOs. Aqueous extracts of *S. canadensis*, *S. virgaurea* and *S. × niederederi* flowers showed the highest inhibitory effect on model plants (*Lactuca sativa* and *Lepidium sativum*) seed germination and growth. In comparison to parental plant species, the hybrid (*S. × niederederi*) also effectively suppressed tested plants’ germination and growth. When comparing model plants it was noticed that garden pepper cress was more susceptible to *Solidago* inhibitory effects than lettuce.

Root and inflorescence EOs of *S. canadensis* showed the highest inhibitory effects on model plant seed germination and growth. It was noticed that lettuce seeds were more susceptible to Canadian goldenrod EO inhibitory effects than garden pepper cress seeds. Allelopathic properties of *S. canadensis* are related with the chemical composition of various extracts and EOs.

This study revealed that it is important to investigate allelopathic mechanisms and to improve our understanding about its role in the spread of invasive species. Furthermore, we recommend to control non-native goldenrods before the flowering period and not leave removed biomass during the restoration process of territories which are colonized by these plants. Obtained data could be important for further regulation and monitoring of the spread of invasive *Solidago* species.

## Figures and Tables

**Table 1:** Conductivity and pH values of soil (n = 3, average mean ± SD, four different sampling sites, I–IV) of *Solidago* spp. growing locality.

Sampling Site	Conductivity, µS/cm	pH Values
I	99.03 ± 0.40–103.83 ± 1.11	6.15 ± 0.07
II	99.07 ± 2.82–116.57 ± 10.10	6.48 ± 0.19
III	92.33 ± 0.55–125.63 ± 6.04	6.65 ± 0.27
IV	95.02 ± 0.55–115.50 ± 2.02	6.45 ± 0.11

**Table 2:** Main elements (weight %, n = 3, standard deviation (SD)) in the soil (four different sampling sites, I–IV) of *Solidago* spp. growing habitat.

Sampling Sites	Ca	Mg	K	Na	Al	Mn	Cu	Cd	Cr	Ni	Pb	Zn	Fe	Mo	P
λ, nm	317.93	285.21	766.49	589.59	396.15	257.61	327.39	228.80	267.72	231.60	220.35	213.86	238.20	202.03	231.67
I	1.232	0.391	0.132	0.009	2.257	0.577	0.023	0.003	0.003	0.001	0.007	0.033	3.513	0.002	0.412
SD	0.130	0.044	0.025	0.007	0.227	0.119	0.005	0.001	0.002	0.000	0.001	0.001	0.483	0.002	0.039
II	3.098	0.587	0.152	0.001	2.148	0.489	0.020	0.000	0.003	0.002	0.007	0.038	3.633	0.000	0.400
SD	0.059	0.046	0.007	0.001	0.085	0.072	0.004	0.000	0.001	0.001	0.002	0.013	0.260	0.000	0.007
III	1.409	0.428	0.152	0.005	2.038	0.412	0.021	0.001	0.005	0.003	0.006	0.024	2.750	0.001	0.300
SD	0.087	0.033	0.013	0.003	0.100	0.041	0.002	0.000	0.003	0.001	0.001	0.002	0.147	0.000	0.018
IV	1.829	0.421	0.150	0.008	2.128	0.442	0.020	0.002	0.004	0.001	0.006	0.034	3.150	0.001	0.350
SD	0.067	0.013	0.011	0.004	0.102	0.051	0.001	0.001	0.001	0.001	0.002	0.002	0.247	0.001	0.022

**Table 3:** Tentative identification of main compounds in methanol/water (MeOH:H_2_O, 70:30 *v/v*) *Solidago canadensis* inflorescence, leaf and root extracts analyzed by HPLC-DAD-TOF.

Identity	t_R,_ min	Molecular Formula	Molecular Mass	Observed *m/z* [M + H]^+^, Da	Observed *m/z* [M − H]^−^, Da
Neochlorogenic acid ^L,F^	7.7	C_16_H_18_O_9_	354.31	355.12	352.97
Chlorogenic acid ^L,F^	8.2	C_16_H_18_O_9_	354.31	355.12	352.96
4-*O*-Caffeoylquinic acid ^L,F^	8.3	C_16_H_18_O_9_	354.10	355.10	353.02
Caffeoylshikimic acid ^R^	8.8	C_16_H_16_O_8_	336.29		335.00
Cinnamic acid ^R^	8.9	C_9_H_8_O_2_	148.16	149.02	147.31
Feruloylquinic acid ^L^	9.7	C_17_H_20_O_6_	368.30		366.99
Rutin ^L,F^	10.0	C_27_H_30_O_16_	610.52	611.16	608.96
Quercetin 4′-(E-6-*O*-caffeoyl)glucoside ^L,F^	10.6	C_30_H_26_O_15_	626.50	627.16	625.39
3,5-Dicaffeoylquinic acid ^L,F^	10.7	C_25_H_24_O_12_	516.45	517.13	514.96
Narcissin ^L^	10.9	C_28_H_32_O_16_	624.50		622.99
Quercetin-3-*O*-(6′′-acetyl glucoside) ^L,F^	11.5	C_23_H_22_O_13_	506.4	507.11	504.94
6′′-*O*-Acetylglycitin? ^L^	11.7	C_24_H_24_O_11_	488.40	488.95	
Ferulic acid ^R^	12.5	C_10_H_10_O_4_	194.18		193.97
Erythrodiol-3-acetate ^R^	13.0	C_32_H_52_O_3_	484.8	485.05	
Isoquercetin (quercetin-3-*O*-glucoside) ^L,F^	14.1	C_21_H_20_O_12_	464.096	468.98	466.93
Caffeoylshikimic acid glucoside ^R^	14.5		499.12	500.93	498.51
Hyperoside (quercetin-3-*O*-galactoside) ^L,F^	15.6	C_21_H_20_O_12_	464.38	465.07	463.25
Cyanidin 3-*O*-glucosyl rutinoside ^F,R^	22.6	C_33_H_41_O_20_^+^	757.77	758.57	756.01
Quercetin caffeoyl hexoside ^F,R^	24.8	C_35_H_34_O_19_	758.2	759.05	757.13
Saponin 1 ^R^	27.9			894.76	
Saponin 2 ^R^	29.9			1112.88	
Cinnamtannin A2 ^R^	35.4	C_60_H_50_O_24_	1155.02	1156.91	

^L,F,R^ Compounds identified in *S. canadensis* leaf, flowers and root extracts, respectively.

**Table 4:** Major (≥3.0%) volatile organic compounds determined in water/diethyl ether *Solidago canadensis* inflorescence, leaf and root extracts of different acidity (n = 3, average mean ± SD, plants collected from four sites of the investigated area).

Compound (RI _Exp_)		Flowers			Leaves			Roots	
	pH = 3.0	pH = 5.9	pH = 11.0	pH = 3.1	pH = 5.4	pH = 11.1	pH = 3.2	pH = 6.3	pH = 11.2
*α*-Pinene * (935)	0.8 ± 0.11	0.5 ± 0.11	1.5 ± 0.47	0.3 ± 0.22	2.5 ± 0.72	0.3 ± 0.21	9.7 ± 1.22	9.5 ± 2.04	10.2 ± 1.41
*β*-Pinene * (978)	0.6 ± 0.28	1.4 ± 0.20	1.5 ± 0.10	0.9 ± 0.61	1.7 ± 0.33	0.5 ± 0.10	5.5 ± 0.45	3.7 ± 0.22	2.0 ± 0.24
*p*-Cymene (1018)	0.6 ± 0.10	0.7 ± 0.33	0.9 ± 0.09	4.4 ± 0.21	1.6 ± 0.18	0.3 ± 0.01	2.3 ± 1.72	2.0 ± 0.37	1.2 ± 0.12
Limonene (1027)				0.5 ± 0.25	3.2 ± 0.44	0.2 ± 0.14	5.2 ± 1.02	3.5 ± 0.72	1.3 ± 0.33
1,8-Cineole * (1033)	0.2 ± 0.01	0.5 ± 0.15	0.7 ± 0.12	1.1 ± 0.01	1.1 ± 3.23	0.3 ± 0.12	11.2 ± 1.33	13.4 ± 0.78	16.1 ± 1.71
*trans*-Pinocarveol (1135)	0.4 ± 0.17	0.3 ± 0.15	0.4 ± 0.12	5.0 ± 0.50	5.5 ± 0.95	6.0 ± 0.70	4.1 ± 1.60	4.2 ± 0.91	4.6 ± 1.46
*trans*-Verbenol (1143)	0.2 ± 0.09	0.9 ± 0.24	1.3 ± 0.54	9.1 ± 1.18	20.0 ± 1.55	24.8 ± 1.69			
*p*-Mentha-1,5-dien-8-ol (1164)				5.9 ± 1.45	0.5 ± 0.15	0.6 ± 0.05			
Borneol (1165)	6.4 ± 0.72	4.5 ± 1.51	4.5 ± 1.78	1.2 ± 1.02	2.2 ± 0.51	2.2 ± 1.53			
Terpinen-4-ol (1174)	0.4 ± 0.11	0.3 ± 0.01	0.1 ± 0.01	1.0 ± 0.50	0.4 ± 0.22	0.6 ± 0.18	6.5 ± 0.75	7.2 ± 1.51	8.8 ± 1.81
*p*-Cymen-8-ol (1183)				0.7 ± 0.27	3.4 ± 0.41	0.5 ± 0.27	4.2 ± 0.95	4.1 ± 1.58	4.0 ± 0.83
*α*-Terpineol 1189	0.8 ± 0.61	0.9 ± 0.33	1.1 ± 0.21	0.4 ± 0.02	0.5 ± 0.03	0.3 ± 0.01	17.6 ± 1.63	19.2 ± 2.30	23.3 ± 2.41
Verbenone (1205)	0.4 ± 0.09	0.3 ± 0.04	0.4 ± 0.11	7.6 ± 1.32	8.7 ± 0.67	9.4 ± 1.13			
*trans*-Carveol (1219)				3.0 ± 1.37	3.4 ± 0.05	4.2 ± 0.73			
4-vinyl-Guaiacol (1310)	11.5 ± 0.19	8.9 ± 0.86	3.0 ± 0.95	0.7 ± 0.09	1.5 ± 0.76	0.7 ± 0.04			
*1,2*-Limonene-diol (1321)				2.1 ± 0.33	2.1 ± 0.27	1.9 ± 1.11	3.9 ± 0.67	4.0 ± 0.18	6.5 ± 1.07
Eugenol (1359)	16.1 ± 1.53	11.9 ± 1.94	12.2 ± 0.33	2.2 ± 0.27	1.7 ± 0.63	1.8 ± 0.98			
*trans*-Myrtanol acetate (1381)				2.0 ± 1.04	4.7 ± 1.78	5.4 ± 1.43			
7,8-Dihydro-3-oxo-*α*-ionol (1704)	8.7 ± 1.64	7.1 ± 1.79	7.8 ± 1.33						

RI _Exp_: Retention indices determined experimentally on the nonpolar column Rxi-5 MS; * Additional identification with reference compound.

**Table 5:** Main chemical composition (≥3.0%) of *Solidago canadensis* inflorescence, leaf and root essential oils (n = 3, average mean ± SD, plants collected from four sites of the investigated area).

Compound ^a^	^b^ RI _Lit_	^c^ RI _Exp_	Flowers	Leaves	Roots
*α*-Pinene *	939	938	21.6 ± 3.25	1.2 ± 0.15	2.6 ± 0.75
*β*-Pinene *	980	984	3.2 ± 0.25	0.2 ± 0.13	26.2 ± 2.23
*β*-Myrcene	991	990	3.0 ± 0.55	0.1 ± 0.01	4.1 ± 0.92
Limonene	1029	1030	10.2 ± 1.55	0.6 ± 0.10	35.0 ± 2.60
*trans*-Pinocarveol	1139	1136	1.1 ± 0.35	4.4 ± 1.50	0.2 ± 0.11
*cis*-Verbenol	1140	1145	0.4 ± 0.23	2.7 ± 0.20	0.1 ± 0.01
*trans*-Verbenol	1144	1146	4.5 ± 1.61	21.3 ± 1.04	0.1 ± 0.04
*o*-Mentha-1,5-dien-8-ol	1164	1165	2.2 ± 1.04	3.0 ± 0.41	0.1 ± 0.01
*p*-Mentha-1,5-dien-8-ol	1166	1170	0.1 ± 0.01	4.3 ± 1.55	0.1 ± 0.01
Borneol	1165	1168	0.1 ± 0.01	4.4 ± 1.01	0.1 ± 0.02
Verbenone	1204	1206	1.7 ± 0.62	12.5 ± 1.53	0.7 ± 0.35
*trans*-Carveol	1217	1215	1.6 ± 0.84	4.5 ± 1.10	0.2 ± 0.15
Bornyl acetate	1285	1290	6.6 ± 0.83	6.0 ± 0.70	0.1 ± 0.01
*β*-Elemene	1391	1393	2.9 ± 0.44	0.5 ± 0.24	3.6 ± 1.01
Germacrene D	1480	1485	15.1 ± 5.35	2.0 ± 1.55	2.0 ± 0.25
Viridiflorol	1590	1594	0.5 ± 0.21	3.0 ± 0.75	0.1 ± 0.02
Germacra-4(15),5,10(14)-trien-1-α-ol	1686	1685	0.5 ± 0.14	4.3 ± 1.10	0.2 ± 0.15
Curlone (Turmerone)	1701?	1698	3.1 ± 1.35		
Lupenyl acetate		2145?	9.8 ± 2.35		
Average Total			96.2 ± 1.52	94.1 ± 2.44	89.7 ± 0.95

^a^ Constituents are listed in order of their elution from a nonpolar DB-5 (which is identical to a Rxi-5 MS) column and compounds are identified by their mass spectra and retention indices on both (polar HP-FFAP and nonpolar Rxi-5 MS) columns; ^b^ RI _Lit_: Kovat’s indices for the nonpolar column DB-5 are taken from the literature [61]; ^c^ RI _Exp_: Retention indices determined experimentally on the nonpolar column Rxi-5 MS; *Additional identification with reference compound; a list of minor (<3.0%) constituents identified in inflorescence, leaf and root EOs of *S. canadensis* is presented in Table S1, Supplementary Materials.

**Table 6:** Calculation of germination rate (GR), relative germination (RG) and vigor index (VI) of *L. sativa* and *L. sativum*.

Index	Equation
Germination rate (GR), %	GR = final number of germinated seeds after 5 days of incubation/20 × 100%
Relative germination (RG), %	RG = GRtr (%)/GRcn (%) × 100 GRtr—mean seed germination for each treatment GRcn—mean seed germination for control
Vigor index (VI)	VI = PL (mm) × GR (%)

## Data Availability

Not applicable.

## Supplementary Materials

The following supporting information can be downloaded at: https://www.mdpi.com/article/10.3390/plants12071421/s1. Table S1. Minor constituents (<3.0%) identified in inflorescence, leaf and root EOs of *S. canadensis*. Table S2. The effect of root aqueous extracts of *S. canadensis*, *S. virgaurea* and *S. × niederederi* on germination rate (GR), relative germination (RG) and vigor index (VI) of lettuce (*Lactuca sativa* L.) and garden pepper cress (*Lepidium sativum* L.) seeds. Table S3. The effect of leaf aqueous extracts of *S. canadensis*, *S. virgaurea* and *S. × niederederi* on germination rate (GR), relative germination (RG) and vigor index (VI) of lettuce and garden pepper cress seeds. Table S4. The effect of inflorescence aqueous extracts of *S. canadensis*, *S. virgaurea* and *S. × niederederi* on germination rate (GR), relative germination (RG) and vigor index (VI) of lettuce and garden pepper cress seeds. Figure S1. Sampling site of plant material of *Solidago* species (*S. canadensis*, *S. virgaurea* and *S. × niederederi*) in Eastern Lithuania (Vilnius, Trakai municipality, Lentvaris). Figure S2. Control samples of *Lactuca sativa* L. seeds (A) and *L. sativa* seeds after treatment of aqueous *S. canadensis* root extract (B). Control samples of *Lepidium sativum* L. seeds (C) and *L. sativum* seeds after treatment of aqueous *S. canadensis* root extract (D).
