# Supplementary material for: Allelopathic Activity of Canadian Goldenrod (Solidago canadensis L.) Extracts on Seed Germination and Growth of Lettuce (Lactuca sativa L.) and Garden Pepper Cress (Lepidium sativum L.)
Source: Plants (Basel). 2023 Mar 23;12(7):1421. doi: 10.3390/plants12071421 (PMC10096748; doi:10.3390/plants12071421)
Supplement: Supplementary file 1 [file plants-12-01421-s001.zip › plants-2241625-supplementary.pdf]

Allelopatic Activity of Canadian Goldenrod (*Solidago canadensis* L.) Extracts on Seed Germination and Growth of Lettuce (*Lactuca sativa* L.) and Garden Pepper Cress (*Lepidium sativum* L.)

Asta Judžentienė, Jurga Būdienė, Linas Labanauskas, Donata Stancelytė and Irena Nedveckytė

Table S1. Minor constituents (< 3.0%) identified in inflorescence, leaf and root EOs of *S. canadensis*

**Minor compounds found in the EOs of *S. canadensis* overground parts (inflorescences and leaves)**
tricyclene, α-thujene, α-myrcene, α-phellandrene, α-, γ-terpinene, *p*-cymene, (*Z*),(*E*)-β-ocimene, *cis*-, *trans*-sabinene hydrate, *trans*-linalool oxide, terpinolene, linalool*, α-campholenal, 6-camphenone, perillene, sabina ketone, *cis*-, *trans*-limonene oxide, pinocarvone, terpinen-4-ol, *p*-cymen-8-ol, α-terpineol, myrtenol, myrtenal, carvone, myrtenyl acetate, perilla aldehyde, perilla alcohol, δ-elemene, α-cubebene, eugenol, α-, β-ylangene, α-, β-copaene, β-bourbonene, β-caryophyllene*, (*E*)-β-farnesene, α-humulene*, *cis*-cadina-1(6), 4-diene, *cis*-muurola-4(14), 5-diene, bicyclogermacrene, α-muurolene, β-bisabolene, β-sesquiphellandrene, α-, γ-, δ-cadinene, α-, β-calacorene, germacrene B, nerolidol, spathulenol, caryophyllene oxide*, salvia-4(14)-en-1-one, *cis*-cadin-4-en-7-ol, *ar*-tumerone, α-, τ-muurolol, α-cadinol, 1,3-dihydrofarnesol, 15-oxy-α-muurolene, phytone, farnesyl acetone, n-hexadecanoic acid, (*E*,*E*)-geranyl linalool and phytol.
**Minor constituents determined in the EO of *S. canadensis* hairy roots**
tricyclene, α-thujene, α-myrcene, camphene, sabinene, α-phellandrene, α-, γ-terpinene, *p*-cymene, *cis*-, *trans*-sabinene hydrate, terpinolene, *p*-cymenene, fenchyl alcohol, *cis*-, *trans*-limonene oxide, pinocarvone, terpinen-4-ol, myrtanal, *p*-cymen-8-ol, α-terpineol, myrtenol, myrtenal, thymol methyl ether, carvone, methyl camphonoate, perilla alcohol, carvacrol, (*E*,*E*)-2,4-decadienal, δ-elemene, α-terpinyl acetate, α-, β-copaene, (*Z*)-jasmone, α-bergamotene, β-caryophyllene*, (*E*)-β-farnesene, α-humulene*, α-, γ-curcumene, β-selinene, α-zingiberene, α-selinene, β-sesquiphellandrene, humulene epoxide II, intermedeol, phytone and n-hexadecanol.

* Additional identification with reference compound.

Table S2. The effect of root aqueous extracts of *S. canadensis*, *S. virgaurea* and *S.* × *niederederi* on germination rate (GR), relative germination (RG) and vigor index (VI) of lettuce (*Lactuca sativa* L.) and garden pepper cress (*Lepidium sativum* L.) seeds.

AQUEOUS EXTRACTS FROM ROOTS						
**Treatments**	**Germination rate, %**					
	S. canadensis		*S. virgaurea*		*S.* × *niederederi*	
	Lettuce	Cress	Lettuce	Cress	Lettuce	Cress
**Control**	96.7	95.00	93.33	96.66	95.00	93.33
**0.1**	90.00	96.66	95.00	91.66	96.66	83.33
**0.5**	91.66	76.66	50.00*	63.33*	85.00	76.66
**1.0**	88.33	70.00*	0.00*	8.33*	81.66	0.00*
**Treatments**	**Relative germination, %**					
	S. canadensis		*S. virgaurea*		*S.* × *niederederi*	
	Lettuce	Cress	Lettuce	Cress	Lettuce	Cress
**Control**	100.00	100.00	100.00	100.00	100.00	100.00
**0.1**	93.07	101.75	101.79	94.83	101.75	89.29
**0.5**	94.79	80.69	53.57*	65.52*	89.47	82.14
**1.0**	91.34	73.68*	0.00*	8.62*	85.96	0.00*
**Treatments**	**Vigor index**					
	S. canadensis		*S. virgaurea*		*S.* × *niederederi*	
	Lettuce	Cress	Lettuce	Cress	Lettuce	Cress
**Control**	3613.36	4303.5	4101.85	4734.73	4107.17	4703.83
**0.1**	2973.00	3576.42	3747.75	6234.41	3201.06	3501.25
**0.5**	4700.63	2922.02	390.83	2829.79	2657.67	3746.12
**1.0**	4943.53	3371.67	0.00	11.11	2497.43	0.00

Asterisk (*) indicates a statistically significant difference compared to the control (G-test, $p<0.05$).

Table S3. The effect of leaf aqueous extracts of *S. canadensis*, *S. virgaurea* and *S. × niederederi* on germination rate (GR), relative germination (RG) and vigor index (VI) of lettuce and garden pepper cress seeds.

AQUEOUS EXTRACTS FROM LEAVES						
**Treatments**	**Germination rate, %**					
	S. canadensis		*S. virgaurea*		*S. × niederederi*	
	Lettuce	Cress	Lettuce	Cress	Lettuce	Cress
**Control**	95.00	91.67	95.00	93.33	93.33	93.33
**0.1**	86.67	86.67	88.33	98.33	93.33	86.67
**0.5**	78.33	85.00	91.67	91.67	88.33	63.33*
**1.0**	80.00	70.00	86.67	96.67	85.00	68.33*
**Treatments**	**Relative germination, %**					
	S. canadensis		*S. virgaurea*		*S. × niederederi*	
	Lettuce	Cress	Lettuce	Cress	Lettuce	Cress
**Control**	100.00	100.00	100.00	100.00	100.00	100.00
**0.1**	91.23	94.55	92.98	105.36	100.00	92.86
**0.5**	82.45	92.72	96.49	98.22	94.64	67.86*
**1.0**	84.21	76.36	91.23	103.58	91.07	73.21*
**Treatments**	**Vigor index**					
	S. canadensis		*S. virgaurea*		*S. × niederederi*	
	Lettuce	Cress	Lettuce	Cress	Lettuce	Cress
**Control**	3560.92	6566.63	4056.5	5590.47	4342.96	5646.46
**0.1**	2049.75	4659.96	3587.67	4131.5	3008.34	4729.29
**0.5**	852.49	1358.58	3012.89	1643.95	2203.83	815.9
**1.0**	548.00	165.67	1451.72	159.5	1137.58	86.55

Asterisk (*) indicates a statistically significant difference compared to the control (G-test, p<0.05).

Table S4. The effect of inflorescence aqueous extracts of *S. canadensis*, *S. virgaurea* and *S.* × *niederederi* on germination rate (GR), relative germination (RG) and vigor index (VI) of lettuce and garden pepper cress seeds.

AQUEOUS EXTRACTS FROM INFLORESCENCES						
	**Germination rate, %**					
**Treatments**	*S. canadensis*		*S. virgaurea*		*S.* × *niederederi*	
	Lettuce	Cress	Lettuce	Cress	Lettuce	Cress
**Control**	86.67	95.00	95.00	93.33	93.33	95.00
**0.1**	83.33	88.33	86.67	81.67	95.00	88.33
**0.5**	85.00	58.33*	86.67	71.67	86.67	0.00*
**1.0**	60.00*	51.67*	71.67	50.00*	83.33	0.00*
	**Relative germination, %**					
**Treatments**	*S. canadensis*		*S. virgaurea*		*S.* × *niederederi*	
	Lettuce	Cress	Lettuce	Cress	Lettuce	Cress
**Control**	100.00	100.00	100.00	100.00	100.00	100.00
**0.1**	96.15	92.98	91.23	87.51	101.79	92.98
**0.5**	98.07	61.40*	91.23	76.79	92.86	0.00*
**1.0**	69.23*	54.39*	75.44	53.57*	89.28	0.00*
	**Vigor index**					
**Treatments**	*S. canadensis*		*S. virgaurea*		*S.* × *niederederi*	
	Lettuce	Cress	Lettuce	Cress	Lettuce	Cress
**Control**	3475.47	6574.00	3477.00	5654.24	3386.32	6194.5
**0.1**	1831.87	2271.55	2444.09	3456.00	3272.75	5198.22
**0.5**	640.33	42.77	2088.75	93.17	1310.16	0.00
**1.0**	126.00	27.56	265.18	30.00	552.76	0.00

Asterisk (*) indicates a statistically significant difference compared to the control (G-test, $p<0.05$).

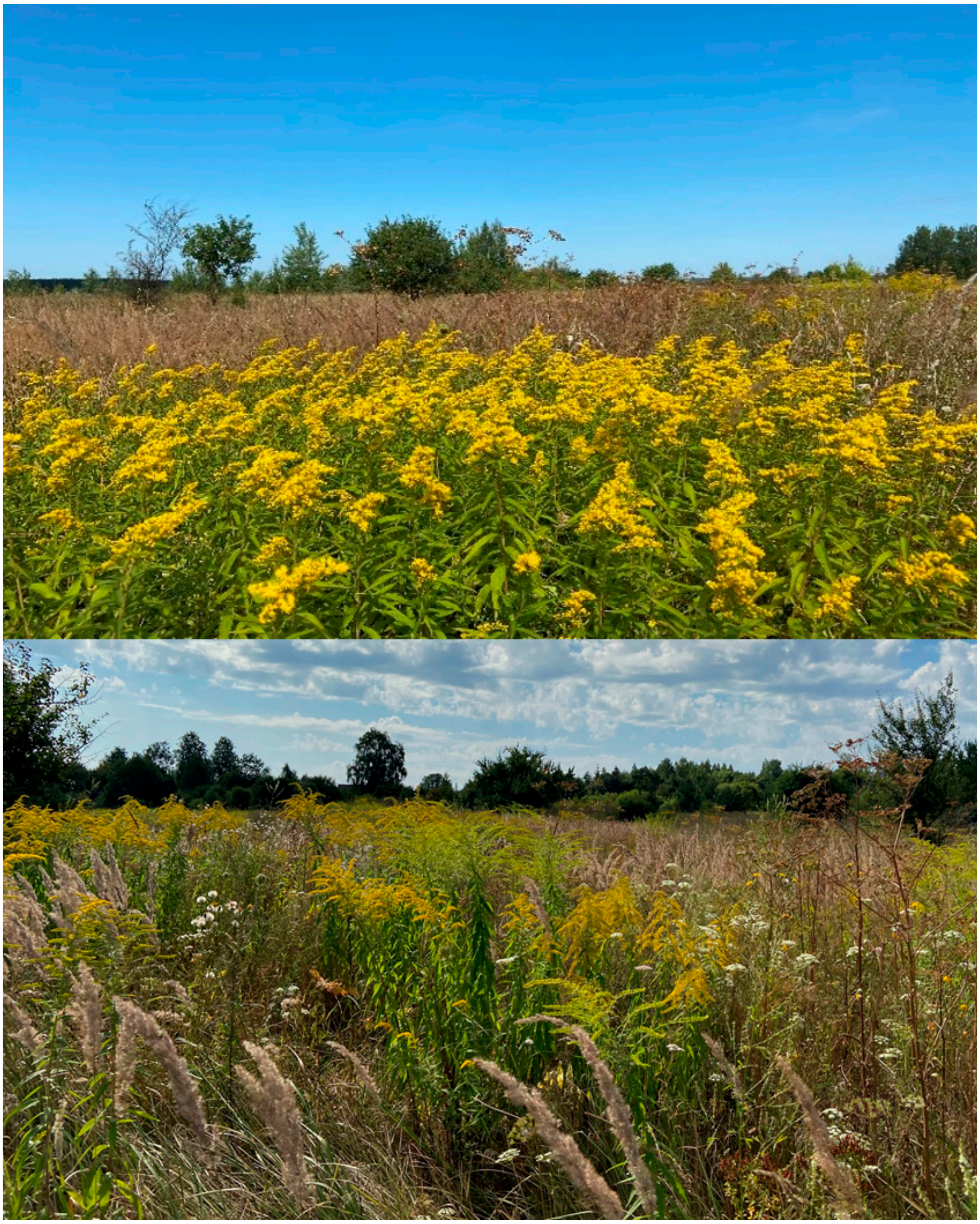

Figure S1. Sampling site of plant material of *Solidago* species (*S. canadensis, S. virgaurea* and *S.* × *niederederi*) in Eastern Lithuania (Vilnius, Trakai municipality, Letvaris).

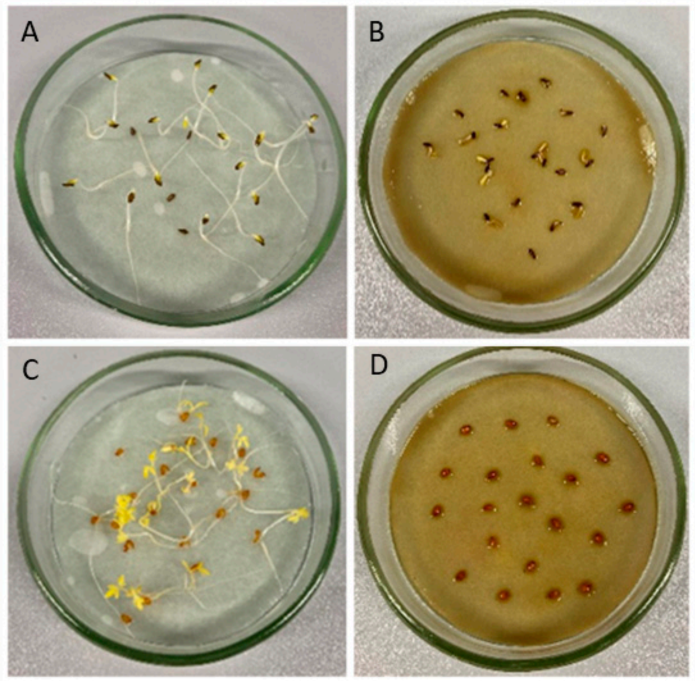

Figure S2. Control samples of *Lactuca sativa* L. seeds (A) and *L. sativa* seeds after treatment of aqueous *S. canadensis* root extract (B). Control samples of *Lepidium sativum* L. seeds (C) and *L. sativum* seeds after treatment of aqueous *S. canadensis* root extract (D).
